# Abnormally high Patient State Index associated with epicardial pacing: a case report

**DOI:** 10.1186/s40981-022-00521-8

**Published:** 2022-05-20

**Authors:** Hiroki Yonezawa, Takuma Maeda, Yoshiaki Takise, Masahiro Morinaga, Yoshihiko Ohnishi

**Affiliations:** Department of Anesthesiology, National Cerebral and Cardiovascular Center, 6-1 Kishibeshinmachi, Suita City, Osaka, 564-8565 Japan

**Keywords:** Patient State Index, Electroencephalography, Monitoring

## Abstract

**Background:**

Anesthesiologists monitor electroencephalography (EEG) intraoperatively to maintain adequate depth of anesthesia. However, the EEG signal is affected by noise and interference. The SedLine® is a brain function monitor with which the Patient State Index (PSI) is calculated. In this study, we report abnormally high PSI values associated with epicardial pacing during open heart surgery.

**Case presentation:**

A 50-year-old man was scheduled for total arch replacement. Atrial demand pacing was started before weaning from cardiopulmonary bypass. The PSI increased from 30 to 80 soon after the start of pacing, and the EEG waveform showed spikes synchronized with the pacing. As the pacing output was lowered, the spikes on the EEG attenuated and disappeared, and the PSI decreased to < 40. When the pacing output was increased again, the spikes recurred, and the PSI increased again.

**Conclusions:**

Pacemaker spikes may cause contamination of the EEG, resulting in abnormally high PSI values.

## Background

Anesthesiologists monitor electroencephalography (EEG) intraoperatively to maintain adequate depth of anesthesia and to prevent intraoperative awareness and overdose of anesthetic agents. Many commercially available devices use EEG spectral information to indicate patients’ levels of sedation. However, in this process, the EEG signal is affected by noise and interference, which must be eliminated. Common interferences comprise electrical factors (e.g., power lines, electrocardiography) and muscle activity (e.g., eye and scalp movements) [1]. Avoiding these effects as much as possible is essential for more accurate EEG monitoring.

The SedLine® (Masimo, Irvine, CA, USA) is a brain function monitor, with which the Patient State Index (PSI) is calculated by analyzing the four-channel EEG information for the frontal region. The PSI is a numerical value that can be used to evaluate the patient's level of consciousness [2]. PSI is reported to be less affected by electronic devices, such as electrocautery, than conventional monitors [3]. In the present study, we report abnormally high PSI values associated with epicardial pacing during open heart surgery.

## Case presentation

**Fig. 1:**
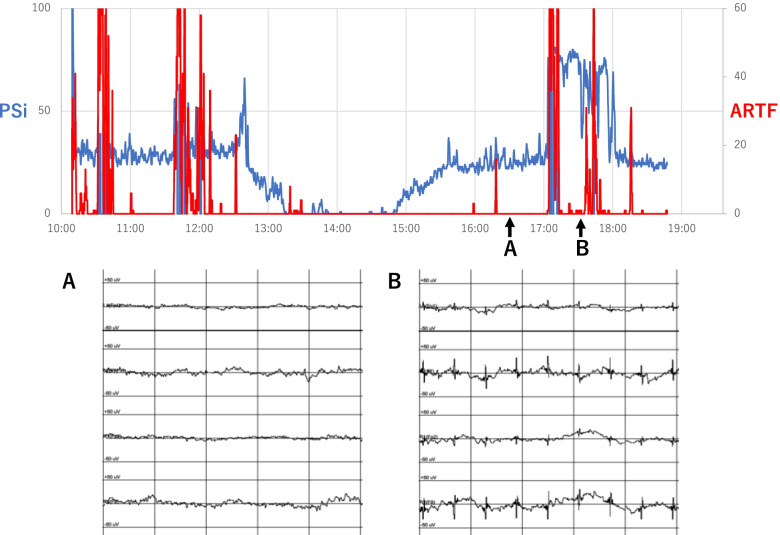
Changes in the Patient State Index (PSI) and electroencephalogram (EEG) after pacing. **A** The EEG wave form without spikes before pacing (PSI: 25, ARTF: 0%, SEFL: 8.0 Hz, SEFR: 4.5 Hz). **B** The EEG waveform with spikes synchronized with the pacing (PSI: 69, ARTF: 0%, SEFL: 7.2 Hz, SEFR: 7.7 Hz). *ARTF*, artifact ratio; *SEF*, spectral edge frequency; *L*, left; *R*, right

Informed consent was obtained from the patient to publish this case report and accompanying images. A 50-year-old man (weight: 96 kg, height: 165 cm) was scheduled for total arch replacement for chronic type A aortic dissection. He had a history of hypertension, dyslipidemia, and putaminal hemorrhage. He had no sequelae of capsular hemorrhage and no history of epilepsy or seizures. Preoperative computed tomography revealed a pseudo-open aortic dissection with an entry in the ascending aorta. There was no extension of the dissection into the neck branches. Chest radiography findings were unremarkable. Blood tests showed a hemoglobin concentration of 16.3 g/dl, creatinine concentration of 1.48 mg/dl, and no electrolyte abnormalities. Electrocardiography indicated normal sinus rhythm, and echocardiography showed no abnormalities in cardiac function or morphology. There were no neurological abnormalities. When the patient was brought into the operating room, his consciousness was clear, and his vitals were stable. A general monitor and the electrodes for PSI (ver. 2310) and regional oxygen saturation (rSO2) monitoring were placed on the patient’s forehead. General anesthesia was induced with 7 mg midazolam, 500 μg fentanyl, and 100 mg rocuronium and maintained with 5 mg/kg/h propofol, 0.5 μg/kg/h remifentanil, and 30 mg/h rocuronium. After induction of anesthesia, the PSI value was 30 (the control PSI value was missing in our data), and the rSO2 value was 60. PSI had hovered near 30 until the start of cardiopulmonary bypass. The total cardiopulmonary time was 270 min, and the circulatory arrest time of the lower body at 26 degrees Celsius was 71 min. During hypothermia, PSI was 0 but increased to 30 as body temperature returned to normal. There was no decrease in rSO2 during the cardiopulmonary bypass. Atrial demand (AAI) pacing was started using alligator clips attached to the epicardium and pleura before weaning from cardiopulmonary bypass. A Medtronic (Minneapolis, MN, USA) 5348 single-chamber temporary pacemaker was used to control the output and pacing mode. The heart rate was set at 100 beats/min, and the pacing output was 20 mA. The PSI increased suddenly to 80 soon after the start of pacing. The EEG waveform showed spikes synchronized with the pacing (Fig. 1), and the artifact ratio (ARTF) ranged from 0% to 20%. To confirm that the AAI pacing waveform influenced the PSI, we lowered the pacing output, as shown in Video 1. As we lowered the pacing output, the spikes on the EEG attenuated and disappeared, and the PSI decreased to < 40. When the pacing output was increased again, the spikes recurred, and the PSI increased accordingly (Video 1). It was noteworthy that PSI was abnormally high while ARTF remained at zero because this means that the SedLine® algorithm could not recognize the pacemaker ECG as noise. After the epicardial pacemaker wire was inserted, we switched to epicardial pacing. The spikes disappeared at that time, and the PSI value decreased to 30. Postoperatively, we confirmed that there were no problems with the administration of anesthetics. There were also no abnormal neurological findings and no episodes of intraoperative awareness.

## Discussion

We experienced a case in which epicardial pacing resulted in an abnormally high PSI value. Previously, there have been reports of epicardial pacing affecting the bispectral index (BIS-XP®; Aspect Medical Systems, Newton, MA, USA) [4]. However, to our knowledge, the same phenomenon has not been reported for PSI. In our case, the increase in PSI and pacing spikes on the EEG were observed immediately after the start of pacing, suggesting that artifacts caused by epicardial pacing affected PSI. In addition, by decreasing the pacing output, the spike waves decreased, and the PSI value decreased accordingly. Therefore, reproducibility was confirmed.

Recent reports have shown that PSI was elevated by oscillation noise from intra-aortic balloon pumping during cardiopulmonary bypass [5] and that pulsation artifact from the superficial temporal artery contaminated the EEG [6]. In our case, spike contaminations were observed in the EEG when AAI pacing was performed using alligator clips between the epicardium and pleura, but no spike-like contaminations were observed when epicardial pacing leads were used. At our institution, we use bipolar pacemaker wires (OSPYKA temporary myocardial leads 66 TC-5, pacing wires, heart wires). In this wire, two copper wires, insulated from each other, form a single wire that runs across the epicardial surface, and the ends of the wires are approximately 8 mm apart. Therefore, it is difficult to contaminate the EEG with an epicardial pacing lead because the current flows over a short distance. When the epicardium and pleura both have alligator clips attached, the current flows over a longer distance, and the possibility of contaminating the EEG increases. However, in most cases, spike contamination of the EEG was not observed even when pacing with alligator clips attached to the epicardium and pleura, which may be related to a patient’s specific impedance. In any case, we believe that the knowledge that pacing may cause EEG contamination will help us address unexpected PSI increases.

One of the features of the SedLine® is the simultaneous display of four EEG waveforms, density-modulated spectral array (DSA), spectral edge frequency (SEF), and ARTF to reduce bias due to artifacts, especially those caused by electronic devices, compared with conventional monitors. However, although the EEG clearly showed a pacing waveform in our case, the ARTF ranged from 0 to 20%, and the SEF ranged from 5 to 10 Hz, causing a dissociation regarding the PSI value. In addition, in a report comparing abnormally high PSI in the older and updated algorithms of the SedLine®, the incidence of abnormally high PSI was significantly lower in the updated algorithm group [7]. However, our algorithm was the latest version (ver. 2310). To the best of our knowledge, this is the first report showing that the SedLine® could not recognize the pacemaker ECG as noise, even with the latest version of the algorithm. To evaluate the depth of general anesthesia, it is necessary to interpret PSI, DSA, SEF, ARTF, and the EEG comprehensively.

## Conclusion

Pacemaker spikes may cause contamination of the EEG, resulting in abnormally high PSI. Anesthesiologists must comprehensively interpret DSA, SEF, ARTF, and the EEG when evaluating the depth of general anesthesia, not only the PSI values.

## Data Availability

Not applicable

## Abbreviations

EEG: Electroencephalography
PSI: Patient State Index
rSO2: Regional oxygen saturation
ARTF: Artifact ratio
DSA: Density-modulated spectral array
SEF: Spectral edge frequency
AAI: Atrial demand pacing
